# Supplementary figures and images for: Zika virus infection drives epigenetic modulation of immunity by the histone acetyltransferase CBP of Aedes aegypti
Source: PLoS Negl Trop Dis. 2022 Jun 27;16(6):e0010559. doi: 10.1371/journal.pntd.0010559 (PMC9269902; doi:10.1371/journal.pntd.0010559)

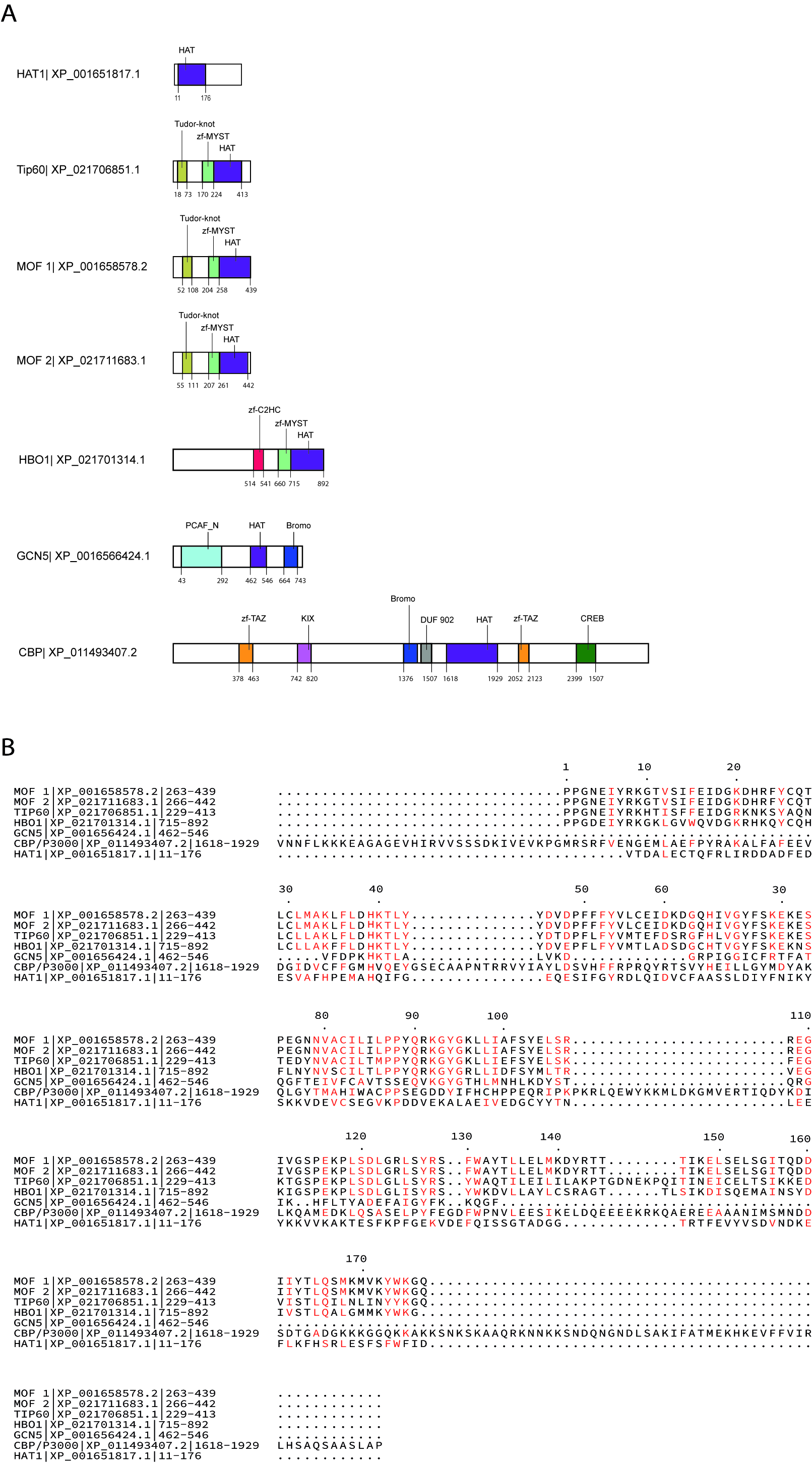

Supplement: S1 Fig — A. Genome identification of putative lysine acetyltransferase (KAT) homologs from A. aegypti, AaHAT1 (XP_001651817.1), AaTip60 (XP_021706851.1), AaMOF1 (XP_001658578.2), AaMOF2 (XP_021711683.1), HBO1 (XP_021701314.1), GCN5 (XP_0016566424.1) and AaCBP itself. Functional domains are indicated above each box. B. Protein sequence alignment of the HAT domains from the putative A. aegypti KATs. Amino acids in red show identity or conservation among all 7 HAT domains. (TIF) [file pntd.0010559.s002.tif]

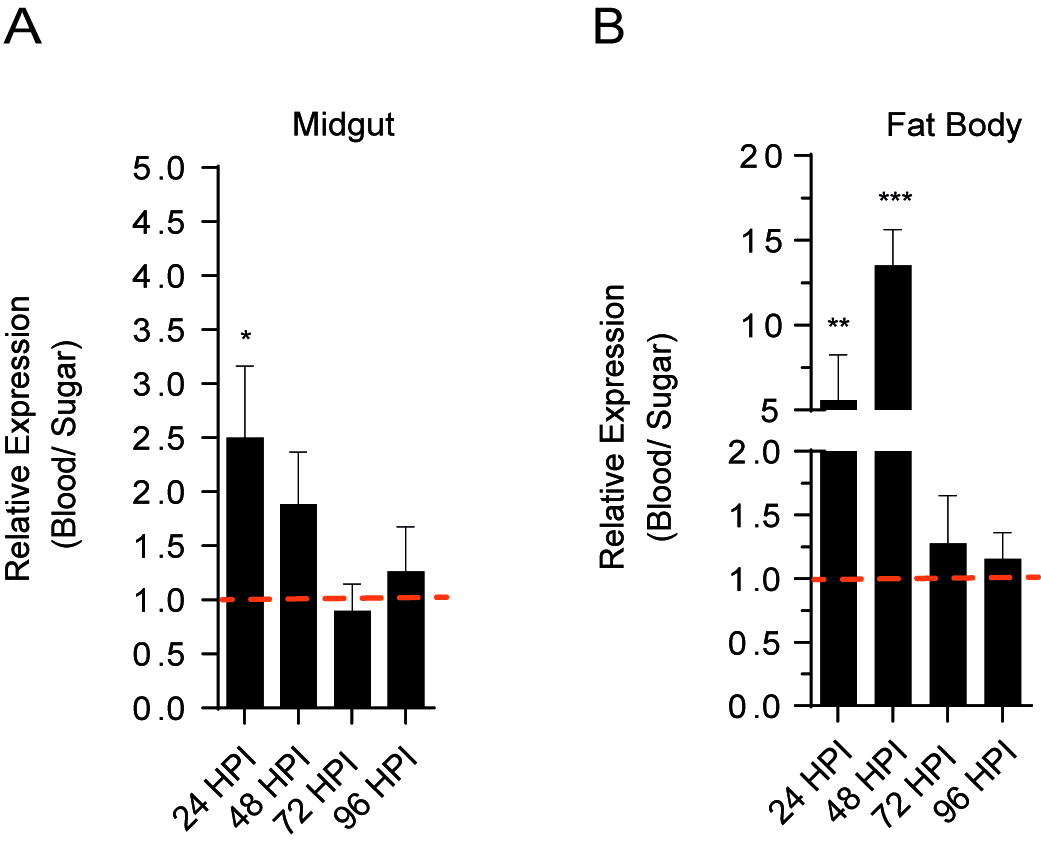

Supplement: S2 Fig — Mosquitoes were fed with blood over different time courses and mRNA quantification by qPCR was performed in the midgut or fat body. The results in A and B are pools of at least 3 independent experiments, plotted using samples from sugar-fed mosquitoes as reference. Error bars indicate the standard error of the mean; statistical analyses were performed by Student’s t test. *, p <0.05; **, p < 0.01; ***, p <0.001. (TIF) [file pntd.0010559.s003.tif]

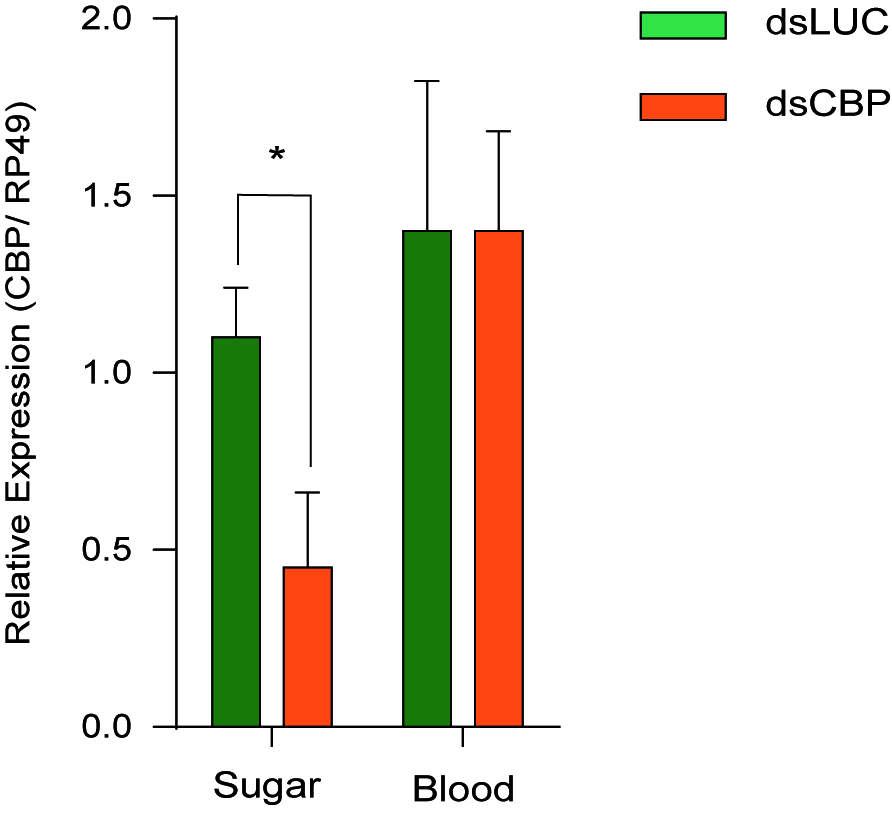

Supplement: S3 Fig — Two days after feeding, the AaCBP gene was knocked down and its expression was measured two days after silencing. qRT-PCR was performed from 3 independent biological replicates. Bars indicate the standard error of the mean; statistical analyses were performed by Student’s t test. *, p < 0.05. (TIF) [file pntd.0010559.s004.tif]

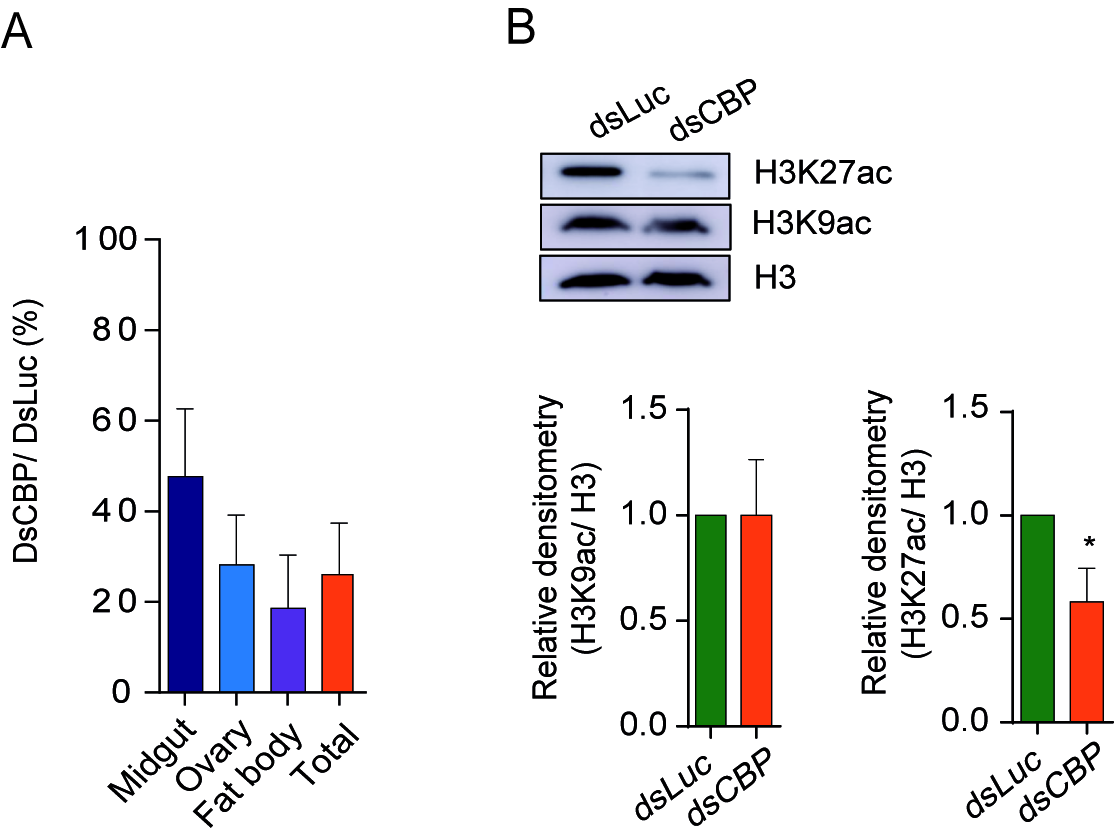

Supplement: S4 Fig — The expression (A) and activity (B) of AaCBP were used to evaluate the efficiency of gene knockdown. A. The mRNA levels of AaCBP in the midgut, ovary, fat body or whole mosquito were quantified by qRT-PCR at 48 h postinjection with dsCBP or dsLuc. Silencing level was determined by the ratio between mRNA levels of AaCBP-silenced versus dsLuc-injected mosquitoes. B. Western blot of 10 μg of total protein extract of dsCBP- or dsLuc-injected-mosquitoes. Monoclonal antibodies against acetylated- or nonacetylated histone H3 (loading control) are indicated. The intensity of the bands was quantified by densitometry analysis plotted as a graph using ImageJ (NIH Software). Western blotting was performed on 3 independent biological replicates and one representative is shown here. Bars indicate the standard error of the mean; statistical analyses were performed by Student’s t test. *, p <0.05. (TIF) [file pntd.0010559.s005.tif]

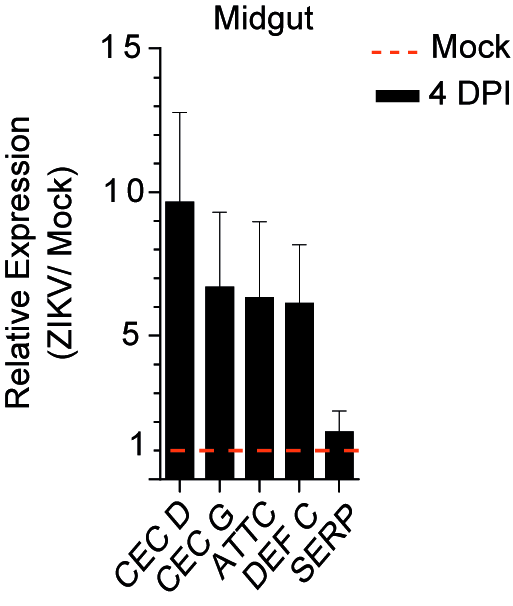

Supplement: S5 Fig — Fifty adult female mosquitoes were infected with ZIKV after feeding on infected blood and the expression of cecropin D (CEC D), cecropin G (CEC G), attacin (ATTC), defensin C (DEF C) and serpin (SERP) was measured by qRT-PCR four days post infection. qRT-PCR was performed from 3 independent biological replicates. Bars indicate the standard error of the mean. (TIF) [file pntd.0010559.s006.tif]

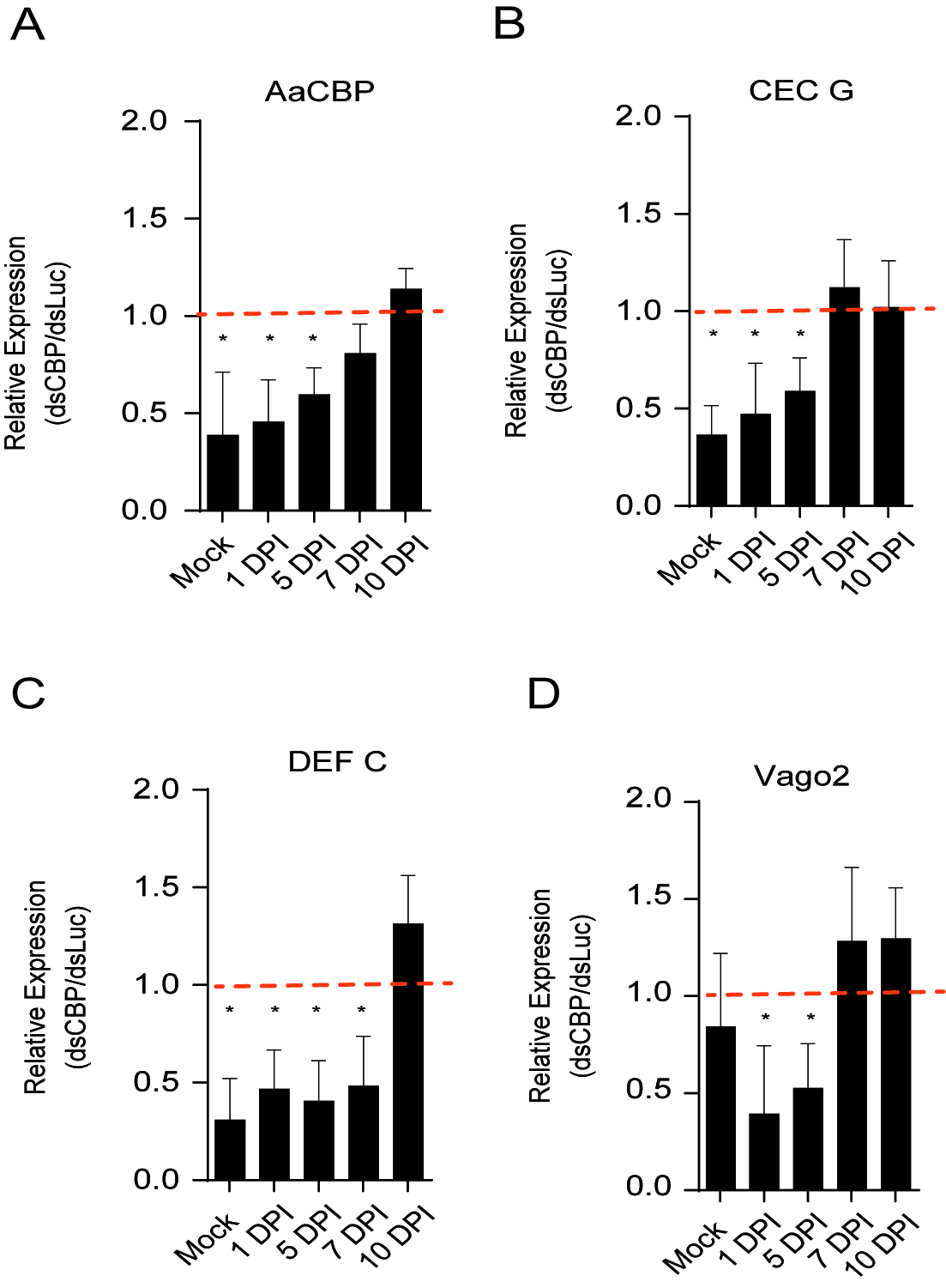

Supplement: S6 Fig — Fifty adult female mosquitoes were infected with ZIKV for 1, 5, 7 or 10 days and the expression levels of AaCBP, cecropin G, defensin C or vago 2 in the fat body were measured by qRT-PCR on 3 independent biological replicates. Bars indicate the standard error of the mean; statistical analyses were performed by Student’s t test. *, p <0.05. (TIF) [file pntd.0010559.s007.tif]

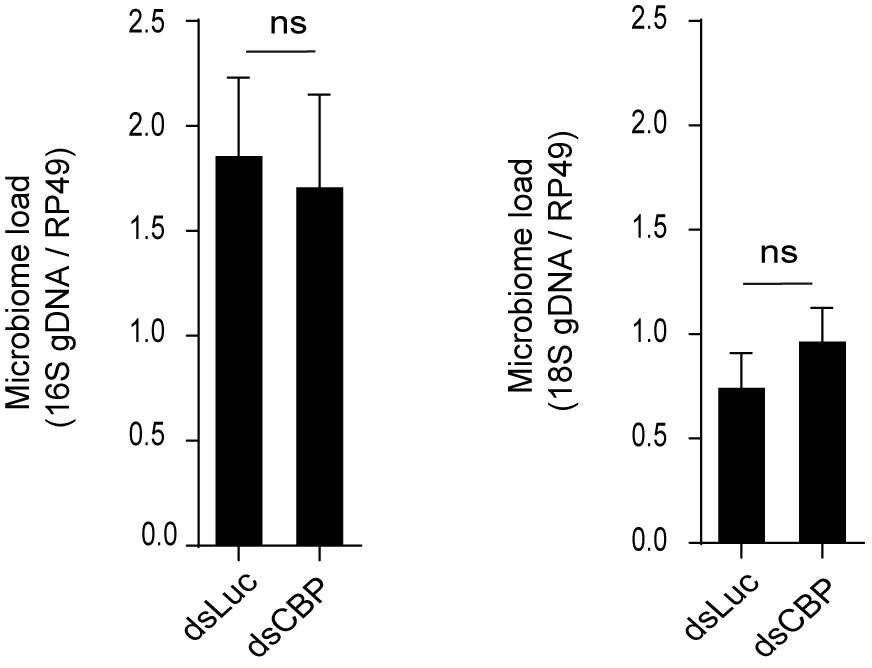

Supplement: S7 Fig — Fifty nanograms of genomic DNA from a pool of 15 midguts from silenced female mosquitoes was used as a template. Amplifications were carried out using specific primers for the ribosomal genes 16S and 18S, for bacteria or fungi, respectively. The A. aegypti ribosomal protein 49 gene (Rp49) was used as an endogenous control. Quantifications were carried out using the comparative Ct method. Genomic DNA qPCR was performed from 6 independent biological replicates. Bars indicate the standard error of the mean. (TIF) [file pntd.0010559.s008.tif]

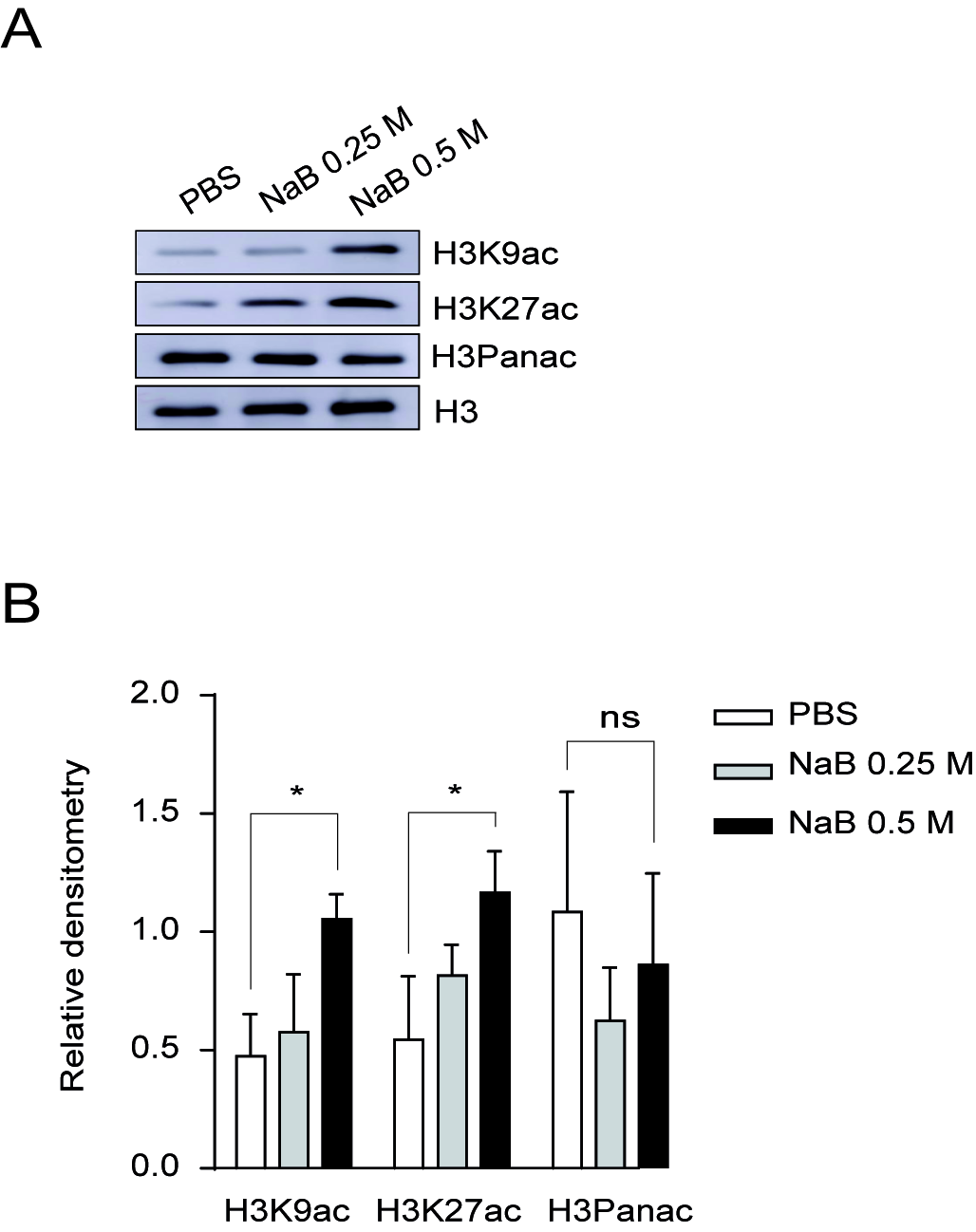

Supplement: S8 Fig — Ten adult female mosquitoes were intrathoracically injected with PBS, 0.25 M (17.5 pmol), or 0.5 M (35 pmol) of sodium butyrate (NaB). Four days after treatment, 10 μg of total protein extract from 10 mosquitoes was used for histone acetylation analysis. Western blotting with monoclonal antibodies against H3K9ac, H3K27ac, H3 panacetylated, or H3 (as loading control) was performed. The intensity of the bands was quantified by densitometry (lower panel) analysis plotted as a graph using ImageJ (NIH Software). Western blotting was performed on 3 independent biological replicates and one representative is shown in panel A. Error bars in Panel B indicate the standard error of the mean; statistical analyses were performed by Student’s t test. *, p <0.05. (TIF) [file pntd.0010559.s009.tif]
