# Supplementary material for: Zika virus infection drives epigenetic modulation of immunity by the histone acetyltransferase CBP of Aedes aegypti
Source: PLoS Negl Trop Dis. 2022 Jun 27;16(6):e0010559. doi: 10.1371/journal.pntd.0010559 (PMC9269902; doi:10.1371/journal.pntd.0010559)
Supplement: Attachment — Submitted filename: Rebuttal letter PNTD-D-21-01702.pdf [file pntd.0010559.s010.pdf]

Instituto de Bioquímica Médica Leopoldo de Meis

Marcelo R. Fantappié, Ph.D.
Associate Professor
Institute of Medical Biochemistry
Federal University of Rio de Janeiro
Prédio do CCS, Bloco Dss - 016
Cid. Universitária – Ilha do Fundão
Rio de Janeiro - RJ - 21.941-902 - Brasil
Tel: +55 21 3938-6608
e-mail: fantappie@bioqmed.ufrj.br

PLOS Neglected Tropical Diseases

Jason L. Rasgon - Associate Editor

Phillipe Desprès – Deputy Editor

RESPONSE LETTER

Rio de Janeiro, April 25th, 2022

Dear Dr. Rasgon and Dr. Desprès,

Please, find below our revised manuscript entitled Zika virus infection drives epigenetic modulation of immunity by the histone acetyltransferase CBP of *Aedes aegypti* by Amarante *et al*., which we would like to be reconsidered for publication in PNTD. Please, find below our point-by-point responses to the reviewers' comments.

We would like to take this opportunity to thank both reviewers for their detailed review of our manuscript. Their inputs were constructive for improving our paper. We hope you will find our revised and improved work suitable for publication in PNTD.

Sincerely,

Marcelo Fantappié

Point-by-point responses to the reviewers' questions (in bold):

Reviewer #1:

In Figure 4 the authors identified three immune genes that are down-regulated in response to dsRNA KD of AaCBP, namely Cecropin D, Cecropin G, and Defensin C. However, in Figure 5 they did not include Cecropin G for analysis and included Defensin A. An explanation or addition of data for Defensin A in figure 4 would bring more clarity for the readers and consistency to the story.

Authors:

We agree. We performed additional qPCR for defensin A and included the data in Figure 4, as well as in the results section (page 14).

Reviewer #1:

In figure 4 legend, defensin is misspelled. Also, to maintain consistency with the other gene names, please change the first alphabet of gene names 'dicer' and 'argonaute' into lower case letters.

Authors:

We corrected it to defensin C and we have changed the gene names, accordingly.

Reviewer #1:

Figure 6 can be presented in a better way e.g., the cytoplasmic and nuclear compartments need to be separated.

Authors:

We agree. We have clarified the nuclear compartment in the figure.

Reviewer #1:

The mechanistic details as explained in the results section, figure legend and discussion are missing in the figure.

Authors:

We believe that the figure, although simple, is clear enough to corroborate our proposed molecular model, which is detailed in the text.

Reviewer #2:

For survival curves the authors report "much higher mortality" for dsAsCBP-ZIKV group, but no statistical analysis is presented.

Authors:

We agree. Statistics are included in Figure 3B (survival curves).

Reviewer #2:

The legend states that 100 mosquitoes were used per group, but no indication is given as to whether this experiment was repeated (or how many times), the size/type of the cage these mosquitoes were held in,

Authors:

All experiments in this study were repeated at least three times. The survival curves were repeated 5 times. This information is provided in the legend of Figure 3B (page 28).

or whether dead individuals were removed on a daily basis. It is not uncommon for mold to grow on the bodies of dead individuals-in a crowded arena this can exacerbate mortality

amongst others in the cage which essentially serves as a feedback loop. This can be mitigated by keeping the mosquitoes in many small groups (5 cups of 20 or even better 10 cups of 10, so an artifactual death spiral would only affect a single replicate cup) or by removing dead individuals each day.

Authors:

Yes, we agree, and we were aware of this potential problem. The survival experiments were conducted in five 500ml cups, each containing 20 mosquitoes. Dead mosquitoes were removed on a daily basis.

Reviewer #2:

The authors show that silencing of AaCBP is over by 10 DPI, and that levels of AMPs and Vago2 are back to normal by 10 DPI (Fig S6). However, differences in ZIKV titer do not emerge until 10 DPI (Fig 3), with no difference at 5 DPI-even though that is when silencing of AaCBP and the AMPs are maximal. This is not consistent with AaCBP or the indicated AMPs acting directly in anti-ZIKV immunity, and this inconsistency is not addressed in the manuscript even though it goes to the heart of the main conclusions. Likewise in Figure 5, NaB experiments show that AMPs are induced in ZIKV-infected individuals at 3 DPI with reduced virus titer at 7 DPI, but not 15 DPI. Again, this is not consistent with the data presented in Fig3, where the authors report an increase in viral titer at 15 DPI after knockdown of CBP.

Authors:

The data of Figure S6 show the levels of mRNA transcripts, which are indeed compromised until Day 5 and start to be restored at Day 10. However, it is difficult to

tell when proteins are fully expressed, translated and/or active to re-establish cellular homeostasis. The same consideration can be assumed for the data of Figure 5.

In addition, please, see the data of Figure 2D and E. Note that in Aag2 cells, AaCBP gene transcripts reach their peak at 6 hours post-infection, but the peak of its enzymatic activity is only reached at 15 hours post-infection.

<u>Reviewer #2:</u>

In reference to Figure 3, the authors state “We clearly saw that AaCBP-silenced mosquitoes did not efficiently fight virus infections in these tissues that are expected to mount strong immune responses against viruses” – This phrase overstates the findings presented. The authors observed a less than 2-fold increase in virus titer at 10+15 DPI. No difference at all was observed at 5 DPI. This does not mean that these mosquitoes did not mount an immune response, and it is not clear what “efficient” means in this context since that is typically a measure of how much you get out based on how much you put in. Also, the term “clearly” is subjective and should be removed. Given that virus titers in the control group here were about 4 times lower at 15 DPI as compared to the control group in Figure 5 (~2000 PFUs per midgut vs 8000 PFUs per midgut), the authors need to be much more cautious in their interpretations since there is clearly substantial variation between experiments.

This variation is never explained at since the authors are injecting virus should not be present (that is the whole point of injections, to make dose uniform).

<u>Authors:</u>

We agree and have changed the overstated phrases.

Experimental variations may occur, but not necessarily during or due to injections; Some variations may occur during plaque experiments, for example. Because of the

different time points, plaque experiments had to be performed on different days, which may account for the observed variations.

Previous studies that also carried out virus injections in *A. aegypti* show significant variations in their data as well. Examples can be found here: 1. Giel P. Goertz et al. PNAS, 2019, Vol. 116, pages 19136-19144; 2. Giel P. Goertz et al. PNTD, 2017, 11(6): e0005654.

Reviewer #2:

The authors state that "An increase in total histone H3 acetylation could not be observed (Supplementary Fig 7A and B), which is somehow expected if one considers that a combination of acetylated and nonacetylated H3 might coexist in a specific cell type and/or during a specific period." – This does not make sense. Acetylation can occur at H3 positions K9, K14, K18, K23 and K27. The authors document what appears to be an increase in acetylation at positions K9 and K27. Total acetylation did not appear to change. Provided the authors data are reliable, the most obvious possibility to check is that acetylation at some of the other positions has decreased. If cell-type specific events were obfuscating total acetylation, they should obscure K9 or K27-specific acetylation as well. Of course, as the authors state, NaB should increase acetylation across all sites, so this is still a bit inconsistent with the authors conclusions.

Authors:

We agree that the H3panac result would not be expected; however, these data were consistent and we wanted to show it. Although we do not have a clear interpretation of this result, we can only speculate at this stage that: 1. The levels of the specific acetylated lysines can vary among the different tissues of the whole mosquito, since we

are analyzing specific marks (K9 or K27) unlike the other five H3 lysine marks (K4, K14, K18, K23 AND K56); 2. In addition, we used a polyclonal antibody for H3panac unlike the monoclonal antibodies used for H3K9ac and H3K27ac, which may account for this discrepancy.

<u>Reviewer #2:</u>

"The Toll pathway has been shown to play the most important role in controlling ZIKV infections (Angleró-Rodríguez et al., 2017)." -This is not an accurate representation of the findings of Anglero-Rodriguez. In that paper, the authors found the strongest anti-viral response when the Toll pathway was artificially stimulated as compared only with IMD and Jak/Stat. These experiments did not consider any other immune pathway, of which there are still many.

<u>Authors:</u>

We agree that our frase is overstated and have changed it accordingly:

The Toll pathway is an NF-*k*B pathway that is <u>suggested</u> to play a role in immunity in mosquitoes (Angleró-Rodríguez et al., 2017; Cheng et al., 2016; Tikhe & Dimopoulos, 2021)".

<u>Reviewer #2:</u>

The authors do not adequately consider other hypotheses that could better explain their findings, making it difficult to come to the same conclusions as are reached in the model presented in Figure 6. Two points that need to be further developed (either experimentally or in the discussion or both) are potential affects on the microbiome and the more general role of CBP outside of its role as a HAT. For the former, dysregulation of AMPs may lead

to dysbiosis or greater susceptibility to fungal pathogens-maybe this can be compensated for alone but not with the added stress of ZIKV infection.

Authors:

We agree and performed an experiment to address the potential effect of the dysregulation of AMPs, leading to intestinal dysbiosis. We carried out 16S (for bacteria) or 18S (for fungi) qPCR of genomic DNA from midguts of dsLuc- or dsCBP-mosquitoes (now Supplementary Fig. 7). We did not observe any significant effect on the microbiota of these mosquitoes. We included these data in the results section, as shown in Supplementary Figure 7 (page 15).

We also performed the same experiment (gDNA qPCR for 16S and 18S) but now with infected silenced-mosquitoes. We observed similar data, with no statistical differences in the microbiome (not included in the paper, but available upon request).

Reviewer #2:

In the latter, dsRNA experiments cannot disentangle the role of CBP in histone acetylation from its more general role as CREB binding protein in RNA transcription.

Authors:

We are not aware of the function of CBP proteins (in all different model organisms thus far studied) outside its role as a transcription coactivator through its histone acetyltransferase activity. However, this is a clear possibility, and this possibility can certainly be addressed in our future studies.
