# Supplementary material for: Zika virus infection drives epigenetic modulation of immunity by the histone acetyltransferase CBP of Aedes aegypti
Source: PLoS Negl Trop Dis. 2022 Jun 27;16(6):e0010559. doi: 10.1371/journal.pntd.0010559 (PMC9269902; doi:10.1371/journal.pntd.0010559)
Supplement: Attachment — Submitted filename: Rebuttal letter PNTD #2.pdf [file pntd.0010559.s011.pdf]

Marcelo R. Fantappié, Ph.D.
Associate Professor
Institute of Medical Biochemistry
Federal University of Rio de Janeiro
Prédio do CCS, Bloco Dss - 016
Cid. Universitária – Ilha do Fundão
Rio de Janeiro - RJ - 21.941-902 - Brasil
Tel: +55 21 3938-6608
e-mail: fantappie@bioqmed.ufrj.br

PLOS Neglected Tropical Diseases

Jason L. Rasgon - Associate Editor

Phillipe Desprès – Deputy Editor

RESPONSE LETTER

Rio de Janeiro, June 1, 2022

Dear Dr. Rasgon and Dr. Desprès,

This is regarding our manuscript PNTD-D-21-01702_R1, entitled Zika virus infection drives epigenetic modulation of immunity by the histone acetyltransferase CBP of *Aedes aegypti* by Amarante *et al*.

Please, find below our point-by-point responses to the comments of reviewer #2. We hope you will find our revised paper suitable for publication in PNTD.

Sincerely,

Marcelo Fantappié

Point-by-point responses to the questions of reviewer #2 (in bold):

<u>Reviewer #2:</u>

The legend states that 100 mosquitoes were used per group, but no indication is given as to whether this experiment was repeated (or how many times), the size/type of the cage these mosquitoes were held in,

Authors:
All experiments in this study were repeated at least three times. The survival curves were repeated 5 times. This information is provided in the legend of Figure 3B (page 28).

New Comment: No information on replicates is present in the legend of Fig3B.

Authors:

The reviewer is correct and we apologize for the missing information. We now included the complete information in the legend of Figure 3 (page 28).

or whether dead individuals were removed on a daily basis. It is not uncommon for mold to grow on the bodies of dead individuals-in a crowded arena this can exacerbate mortality amongst others in the cage which essentially serves as a feedback loop. This can be mitigated by keeping the mosquitoes in many small groups (5 cups of 20 or even better 10 cups of 10, so an artifactual death spiral would only affect a single replicate cup) or by removing dead individuals each day.
Authors:
Yes, we agree, and we were aware of this potential problem. The survival experiments were conducted in five 500ml cups, each containing 20 mosquitoes. Dead mosquitoes were removed on a daily basis.

New Comment: This is re-assuring, but this information is not included in the revised manuscript

Authors:

The reviewer is correct and we apologize for the missing information. We also included this information in the legend of Figure 3 (page 28).

<u>Reviewer #2:</u>

The authors show that silencing of AaCBP is over by 10 DPI, and that levels of AMPs and Vago2 are back to normal by 10 DPI (Fig S6). However, differences in ZIKV titer do not emerge until 10 DPI (Fig 3), with no difference at 5 DPI-even though that is when silencing of AaCBP and the AMPs are maximal. This is not consistent with AaCBP or the indicated AMPs acting directly in anti-ZIKV immunity, and this inconsistency is not addressed in the manuscript even though it goes to the heart of the main conclusions. Likewise in Figure 5, NaB experiments show that AMPs are induced in ZIKV-infected individuals at 3 DPI with reduced virus titer at 7 DPI, but not 15 DPI. Again, this is not consistent with the data presented in Fig3, where the authors report an increase in viral titer at 15 DPI after knockdown of CBP.

Authors:
The data of Figure S6 show the levels of mRNA transcripts, which are indeed compromised until Day 5 and start to be restored at Day 10. However, it is difficult to tell when proteins are fully expressed, translated and/or active to re-establish cellular homeostasis. The same consideration can be assumed for the data of Figure 5.
In addition, please, see the data of Figure 2D and E. Note that in Aag2 cells, AaCBP gene transcripts reach their peak at 6 hours post-infection, but the peak of its enzymatic activity is only reached at 15 hours post-infection.

New Comment: A delay between RNA levels and protein levels may be able to explain a difference of hours, but not days. AMPs by their nature must be produced rapidly and then vanish when the threat is gone. No matter what the explanation, the manuscript must reflect these inconsistencies, rather than ignore them.

Authors:

We agree and included some discussion about this issue in the Discussion section; page 18, lines 10-16.

Reviewer #2:

In the latter, dsRNA experiments cannot disentangle the role of CBP in histone acetylation from its more general role as CREB binding protein in RNA transcription.
Authors:
We are not aware of the function of CBP proteins (in all different model organisms thus far studied) outside its role as a transcription coactivator through its histone acetyltransferase activity. However, this is a clear possibility, and this possibility can certainly be addressed in our future studies.

New Comment: I am confused by this response, as the authors themselves describe CBPs as being "recruited to promoters by interaction with DNA-bound transcription factors, which directly interact with the RNA polymerase II complex.". Thus, CBP is a physical scaffold protein, and the authors also state "The TAZ, KIX and CREB are the protein interaction domains that mediate interactions with transcription factors". Thus, silencing of CBP depleted not just the HAT function, but also the scaffold function. This should be discussed.

Authors:

We agree. The mechanistic action of CBP proteins pointed by the reviewer is correct. We clarified it by including more details about this issue in the text (page 18, lines 10-16).

Reviewer #2:

New comment:
“These results indicate that even a partial deletion of AaCBP is enough to disrupt the homeostasis of the mosquito”- The phrase “partial deletion” here does not make sense, as nothing was deleted. Replace with “partial reduction”, “partial silencing” or similar phrase. Maybe the authors meant “partial depletion”?

Authors:

We agree. We replaced with “partial reduction”. Page 13, line 11).
